# An Optimal Round-Trip Route Planning Method for Tourism Based on Improved Genetic Algorithm

**DOI:** 10.1155/2022/7665874

**Published:** 2022-08-28

**Authors:** Sha Cao

**Affiliations:** College of Land and Tourism, Luoyang Normal University, Luoyang 471934, Henan, China

## Abstract

The optimization of the travel route for a round-trip is not only a customized demand made by a large number of independently guided tourists but also an essential practical issue for the development of tourism management and tourism businesses. In light of this, the study presents a genetic algorithm (GA) as a potential solution to the problem of how to visit a number of tourist destinations within a constrained area in order to quickly determine the shortest tourist route. To traverse areas or regions with the least amount of physical exertion, select the correct and shortest route. Examining all potential routes from the starting point to the destination will allow you to determine the quickest route. A condensed explanation of the enhanced GA is provided to start. The second step is to analyze the model's construction and solution in detail. Next, an enhanced genetic algorithm (IGA) is utilized to determine the optimal travel route for visiting a variety of tourist attractions. In accordance with the optimal travel route, the required number of days and specific travel arrangements are then estimated. In conclusion, the GA is optimized, and a simulation examination of each individual's average path convergence is conducted. The results of the experiments indicate that the IGA can be effectively applied to the path planning of multiple scenic locations, the selection of the shortest travel route, the reduction of travel expenses, and the saving of travel time. This has important implications for both research and practical applications, as well as a high research significance and practical value.

## 1. Introduction

As people's standard of living continues to rise, more of them will choose to travel in their spare time. This trend has contributed to the expansion of the tertiary sector in a number of cities [1]. If you do not plan the route you will take when traveling to an unfamiliar destination in advance, you may encounter complications, such as a longer and more expensive journey. There are numerous requirements that must be met prior to grocery shopping, for instance. Therefore, how to create ideal travel routes based on the specific needs of visitors [2–6] and how to select appropriate travel methods for them in order to enhance their travel experience and improve the quality of life for the locals. Improvement is significant. As a result of the expansion of the national economy, an increasing number of households now own their own automobiles, and the vast majority of people choose to drive when they go on vacation. Currently, a significant number of people are utilizing the Internet. Now, the general public can easily share their inspection records, photographs, and travel experiences. The greater the number of individuals who share their travel experiences on social networks, the greater the quantity of data pertaining to travel. By conversing with others about their travels, one can easily learn about the travel experiences of others and gain crucial information for their own travel plans. However, due to the increased likelihood of traffic congestion during the holiday season, it is especially important to plan your route before setting out. A trip that has been meticulously planned can reduce travel time and expenses, resulting in fewer emissions from the involved vehicles. The optimal path problem studied in this work has the potential to be utilized in additional domains.

The traveling salesman issue [7–9] is the problem of finding a path with the least total of values after traveling to multiple locations and then returning to the point where the journey began. This problem requires visiting all locations, and each location can only be traversed once (the starting point twice). The traveling salesman problem has *N*! possible solutions according to combinatorial analysis. The number of possible combinations will expand at an exponential rate in proportion to the number of cities, *N*. Therefore, employing the exhaustive method will result in a significant increase in the amount of time required. The problem cannot be solved in a time range that is feasible by a typical computer. The GA is able to quickly and easily find the best solution to even the most difficult combinatorial problems, and it can then implement that answer.

The hotel and accommodation sector, the transportation sector, and the entertainment sector, which includes tourist attractions like museums, amusement parks, and sports facilities, among other types of establishments, are all included in the activities that comprise tourism. The actions involved in tourism can be conceptualized as a system that comprises a variety of distinct processes and protocols. Activities related to tourism at the industrial level include planning, organization, and coordination at all levels, as well as training, monitoring, and evaluation at all levels (international, national, regional, anf local). In addition, there is not a singular definition of tourism, and it is challenging to locate a definition of tourism that encompasses all facets of tourism. According to data provided by the OECD in 1991, the idea of tourism is one that may be construed in a variety of various ways depending on the circumstances.

Since the turn of the century, the GA [10–13] has been going through a fruitful phase of development, and it has made its way into virtually every area of mathematics and engineering. Comparable to the traveling salesman problem in the standard path planning problem, the challenge of designing the topology of a distributed computer network, and the challenge of scheduling the production of a workshop. In mathematics, there is also the issue of determining how to get the extreme value of a function that has multiple variables. Obviously, it is also beneficial in the technology that generates natural language, which, among other things, may automatically write some straightforward poems. Even painting and composing, as well as board games like Go and backgammon, fall within the category of more entertainment-focused professions. The GA is an evolutionary concept that models how nature selects the strongest and healthiest individuals to survive. It does this to prevent the GA from getting stuck in a local optimum by mapping the entire search space onto the genetic space. The fitness of the individuals in the population is sorted according to size, and the individuals in the top 40% of the population are copied twice. The individuals in the middle 20% of the population make a copy, while the ones in the bottom 40% do not create a copy.

The traditional traveling salesman problem served as the basis for the tourist route planning problem. Its primary objective is to develop a methodology that, when adhered to, will result in the optimal arrangement of beautiful areas within candidate cities. This issue is currently the subject of a substantial amount of research in both the United States and abroad. The dynamic programming algorithm [14], the simulated annealing technique [15], and the ant colony algorithm [16] are additional methods that have been enhanced. The research conducted by a number of academics demonstrates that route planners give primary consideration to two objectives: achieving the lowest cost and the greatest benefit. This prevents the algorithm from slipping into a local maximum and accelerates its convergence. This work provides a model for planning optimal round-trip routes based on an enhanced evolutionary algorithm. On the basis of previous research, the model was formulated. By adjusting the controllable precision to expand the search range near the end of the algorithm, the risk of missing a superior solution can be reduced. By enhancing the local search capability by introducing screening criteria during the improvement of a suboptimal solution, the convergence speed can be increased while maintaining accuracy. By adjusting the controllable precision to expand the search range near the end of the algorithm, the risk of missing a superior solution can be reduced. By modifying the controllable precision to increase the search area when a penalty factor is introduced, the fitness of an abnormal solution decreases significantly. As a result, the abnormal solution is removed during the update of the subgroup, and its processing is completed in time to make the algorithm's operations run faster.

Moving forward, the subsequent chapters will be organized in this manner. In the second section, the emphasis is on describing the work associated with algorithms, such as determining the most efficient travel routes. In the third section of the article, the introduction of data sets and improved GAs is covered. The fourth component consists of evaluating the outcomes of our model simulation. The fifth and final section is dedicated to conclusion.

## 2. Related Work

There are currently two categories of methods for providing travel recommendations: those that provide recommendations based on the destination and those that provide recommendations based on the route. In the first approach, the emphasis is placed on recommending a single location that offers the optimal combination of features. A frequent example of this is personal travel, which travel agencies use to assist customers in locating the most suitable vacation packages based on the criteria they have specified. Once the process of generating recommendations has been completed, the user is presented with a list of options, each of which has been assigned a rating. The second method not only provides a list of locations that are more relevant to the user's interests but also it assists passengers in planning multistop itineraries. There will always be a demand for a system that can recommend vacation destinations based on the interests and affinities of individual users, given the difficulty of trip planning. Some academics recommend personalized trip itineraries based on user preferences using geotagged photographs. In addition, users have the option of submitting their personal preferences in an interactive format.

There has been an increase in interest in receiving personalized travel advice [17–21]. There are three primary strategies that are frequently utilized in personalized trip suggestions, and these are collaborative filtering, Markov chains, and matrix factorization. Location-based collaborative filtering [22, 23] is one of the most well-known methods for measuring the similarity of social users based on the co-occurrence of previously visited locations of interest, and for then ranking locations of interest based on the visit records of users who are similar to the user being measured. Some academics make use of the model in conjunction with Gaussian density estimation in order to make trip recommendations based on user votes that are similar. Some researchers have constructed a matrix of user locations by utilizing user check-in information locations and a multicenter Gaussian model using the model's probability to analyze users' check-in behavior, including check-in frequency in space, for the purpose of predicting unknown frequencies of these unvisited locations. This matrix of user locations can be used to predict unknown frequencies of these unvisited locations. The independent location model of the user's content preference and the location-aware model of the user's location preference for the tourist point are motivated by the fact that the user's preference for the content of the tourist point is distinct from the user's preference for the tourist point itself. However, when there are few location records of users, location-based approaches may confront the problem of data sparseness. Hence, mining comparable users may be an extremely challenging task. For example, author-topic model-based collaborative filtering approaches simultaneously mine categories of users' topic interests (such as culture, cityscape, landmarks) to facilitate comprehensive recommendations for social users. Topic models are effective at solving problems with limited amounts of data. An iterative learning model that is taught from the authors' offline modules is used by the authors of one paper to present a location and preference-aware trip recommendation system. The method makes use of a weighted category hierarchy to model each person's individual preferences.

A few academics were able to successfully develop tourism routes within the Golden Triangle of the Yellow River by adjusting and refining the parameters of the ant colony algorithm. Some researchers have developed a mathematical model for the planning of the urban domestic waste recycling route, with the objectives of total route length, total vehicle cost, and penalty cost [24]. In addition, these researchers have enhanced the fundamental hybrid leapfrog algorithm and proposed a route planning method for efficiently achieving urban waste recycling. Some researchers have proposed an enhanced hybrid leapfrog algorithm to address the algorithm's slow iteration speed. This algorithm improved the algorithm's evolution speed and accuracy, and it was used to optimize the parameters of the artificial potential field to enhance the path planning capability. Some researchers proposed a mixed integer nonlinear programming model and a genetic algorithm (GA) to solve the model, thereby resolving the issue of flexible planning of the sidewalk network. This model considered the effect of the travel population on the link time and its distribution across the network. Some academics have proposed a meta-heuristic-based method that combines a biased randomization strategy within a variable neighborhood search framework in order to find solutions to multiple problems simultaneously while taking economic, environmental, and social dimensions into account. This method aims to enhance the way in which the search process is directed. There is an issue with the site's vehicle routing. Certain academics [25, 26] have presented a broad variable neighborhood search method in an effort to find a solution to the open vehicle routing problem that minimizes the total number of routes, the total travel cost, and the most time-consuming route.

How can someone, given a limited amount of time, select one of the scenic spots based on their individual needs, return to the starting point without repeating the other scenic spots, and find the shortest and most economical route among all the possible routes? This is an issue that arises when there are many tourist attractions. A tourist route that creates the problem of the itinerant salesperson. Domestic scholars have utilized behavioral geography theory extensively in route planning and optimization in recent years. This is particularly true in a series of microscale studies, and the results have been quite impressive. This pertains to a standard combinatorial optimization issue. Academics have successfully utilized big data from tourist surveys to investigate how behavioral geography theory can be applied to the planning and design of tourist routes, for instance. The connection between the development of travel agency routes and tourist attraction itineraries and operations research has been addressed by a number of academics. Using the scientific principles of graph theory, a select group of researchers was able to construct the optimal tourist itineraries. These researchers also investigated and elaborated on the potential applications and limitations of their research on the design of tourist routes. Few researchers have successfully applied the least spanning tree algorithm [27] to the field of tourism traffic optimization and route organization, resulting in the construction of three themed tourism routes. Using neural network techniques, the traveling salesman problem has been successfully addressed on multiple occasions. Certain researchers transform and normalize the coordinates of Xi'an's tourist attractions and employ the Hopfield neural network approach to create the most efficient tour sequence planning for the city's tourist attractions. A group of researchers created a Hopfield neural network model for Dalingshan Forest Park using criteria such as scenic spot scores, entrance and exit settings, and the shortest paths. In addition, they designed a unique tourism route that incorporates leisure and health care, outdoor experience, and seasonality.

## 3. Research Design

GA is a method for modeling the natural process of biological evolution. It is through the process of population evolution that, in accordance with the theory of natural selection, the best possible answer can be attained.

### 3.1. Data Sources

This section provides a representation of the data set used in the experiments as well as a summary of the results. Using the Open Data Portal, we made these publicly available statistics accessible to the general public. These statistics cover more than 130 competing locations that these visitors visited. It includes a record of each visitor's route during a specific month of the year. Every route consists of a starting point, an ending point, and a number of intermediate travel destinations in between.

In order to train the model, 75% of the data set is utilized as the training set. The trained model is evaluated using a test set consisting of 25 percent of the entire data set. After that, we evaluate the model's overall performance by examining its precision, recall, and precision.

(1) $$\begin{array}{c} \text{Accuracy}=\frac{\text{TP}+\text{TN}}{\text{TP}+\text{TN}+\text{FP}+\text{FN}}\text{,}\\ \text{Precision}=\frac{\text{TP}}{\text{TP}+\text{FP}}\text{,}\\ \text{Recall}=\frac{\text{TP}}{\text{TP}+\text{FN}}. \end{array}$$

### 3.2. GA Algorithm

The following is how the solution process works for the GA algorithm: initially, each target is encoded, and the order in which each code appears in the gene sequence is considered to be an individual. Following the completion of the code, a population of a predetermined size will be randomly formed. Within the context of the optimization process, the fitness function serves a vital purpose. The purpose of determining the fitness function using the shortest travel route throughout the process of optimizing tourist routes is to accomplish the following goals:

(2) $$\begin{array}{c} f=\frac{1}{r_{d}}. \end{array}$$

After identifying the fitness function, start carrying out multiple selection, crossover, and mutation operations on the population. After doing so, calculate the fitness of each individual, and then output the person who has the highest fitness, which is the best travel path. The purpose of the mutation operation is to sporadically alter the location of genes in people within the population. The crossover mutation, the reverse mutation, and the switch mutation are all examples of common mutation operations. The following procedures are required in order to carry out reverse mutation:

  Step 1. Choose two different insertion places at random.
  Step 2. To obtain the offspring, one must first invert the coding order of the portion of the two insertion points that is located in the middle.

### 3.3. IGA Algorithm

The General Accounting Office uses natural number coding, binary coding, and gray code coding. The natural number coding consists of 1, 2, ..., *n*, and since the number 0 is required to split each travel route in order to represent a large number of travel routes, the natural number coding consists of natural numbers. The binary code consists only of the numbers 1 and 0, making it a relatively simple system. It has the advantages of straightforward and simple encoding and decoding operations, as well as easily implemented genetic operations, such as crossover and mutation, and it adheres to the principle of minimum character set encoding. The path optimization problem is an order-based combinatorial optimization problem. Using the Gray code, there is only one bit of difference between the encoded values that correspond to two consecutive integers.

The Gray code method improves the GA's local search capabilities. Additionally, the crossover mutation is simple to implement. And when natural number coding is considered alongside the research problem presented in this paper, it is determined to be the optimal solution.

Utilizing the coding of chromosomal natural numbers, it is possible to guarantee that the rider has a clear driving route. However, it cannot guarantee that each decoded route meets the load star constraint and the time frame constraint. This section describes the function of the punishment.

(3) $$\begin{array}{c} f\left(t\right)=c\left(t\right)+\lambda q\left(t\right)+\gamma w\left(t\right). \end{array}$$

Among these are the following: *c*(*t*) represents the distance that tourists travel, *q*(*t*) represents the sum of the passenger capacity limitations of each route, and *w*(*t*) represents the sum of the sightseeing time limits of each route. Because the restriction on passenger capacity is less severe than the restriction on sightseeing time along the route, *λ*  = 10, *γ*  = 100.

(4) $$\begin{array}{c} w\left(t\right)=\sum_{i=1}^{n}\text{max}\left\{\left(a_{i},b_{i}\right),0\right\}. \end{array}$$

Among them, *a*_*i*_ stands for the amount of time that tourists are expected to spend traveling, while *b*_*i*_ illustrates the actual amount of time that tourists spend traveling.

Data normalization is required. For the purpose of data normalization, this study plans to employ the following formula.

(5) $$\begin{array}{c} X_{N}=\frac{X}{X_{0}}, \end{array}$$

where *X*_0_ is the value of *X* when it is first calculated.

The fitness function *F*(*t*) is defined as the inverse of the function *f*(*t*), which is defined as follows:

(6) $$\begin{array}{c} F\left(t\right)=\frac{1}{f\left(t\right)}. \end{array}$$

Due to the maximum passenger capacity limit, the generation of travel routes requires two processes during the startup phase. These are the processes involved: Set the number of distribution routes to *k* = 1, and once the first route reaches the required passenger capacity, the time window will determine the sequence number of those who have passed through the area. Fill the path after storing the first *k* paths that satisfy the passenger capacity constraint. If the kth path does not meet the required passenger capacity, update *k* to *k *+* *1 after recording the first *k* paths.

After all of the travelers have passed through, they will obtain multiple paths, connect each path with 0, and then obtain a chromosomal number containing all of the client serial numbers. This chromosomal number represents a comprehensive itinerary for a round trip. The appropriate number of chromosomes will be produced when the population size is set to 100. At this point, all necessary steps have been taken to initiate the population.

The GA's selection operation can only eliminate individuals with a superior population and pass them on to the offspring population. This process will not result in the formation of any new individuals. It is necessary to employ crossover in order to preserve the population's genetic diversity, prevent prematurity, and achieve global optimization as efficiently as possible. And mutation operation, the former of which is to randomly swap a portion of the genes of two individuals in a population, and the latter of which is to randomly mutate a portion of the genes of an individual, so that both operations generate new individuals and improve the population. In addition to selecting the appropriate crossover and mutation methods, the GA's crossover and mutation operations must set the probability value and the mutation probability value. This is because the latter has a very obvious effect on the algorithm's global optimization effect.

The selection process is carried out using a method known as the roulette method, which is sometimes referred to as the proportionate selection algorithm. The fundamental notion behind this is that the probability of an individual being chosen for further development increases proportionately with their fitness value. The following are the specific measures to take:

**Step 1 .**

It is to compute the fitness value of each individual based on formula (6).

**Step 2 .**

Use the appropriate formula to determine the likelihood that each individual will be passed down to the subsequent generation (7).

(7) $$\begin{array}{c} p\left(s_{i}\right)=\frac{f\left(s_{i}\right)}{\sum_{i=1}^{n}f\left(s_{i}\right)}, \end{array}$$

*i* = 1, 2, ..., *n*, where *n* is the total number of people in the population.

**Step 3 .**

Perform the following calculation to get the cumulative probability of each individual:

(8) $$\begin{array}{c} q_{i}=\sum_{i=1}^{n}p\left(s_{i}\right). \end{array}$$

The cumulative probability of individual I is denoted by the symbol *q*_*i*_.

**Step 4 .**

In the interval [0, 1], generate a random number *m*, and if *m* < *q*_1_ is found, select individual 1, else select individual *k*, so that *q*_*k*−1_ < *m* < *q*_*k*_ is found.

**Step 5 .**

Repeat the previous step a number of times.

Alternate between the chosen parents in order to produce new offspring.

After performing the crossover operation on the chromosomes, first establish the gene crossover points. The gene crossover points of parent 1 are 3, 4, 5, 6, 7, and the gene crossover points of parent 2 are 7, 6, 5, 4, 3, after performing the crossover operation on the chromosomes. The progeny chromosomes obtained by the operation contain duplicate chromosomes. Therefore, the deletion operation is performed, and the progeny chromosomes are finally obtained, thereby completing the operation of exchanging chromosomes. The progeny chromosomes obtained by the operation contain duplicate chromosomes.

The operation of mutation is not very complicated. The offspring are produced by first selecting two gene locations from each parent at random and then exchanging those positions.

**Figure 1:**
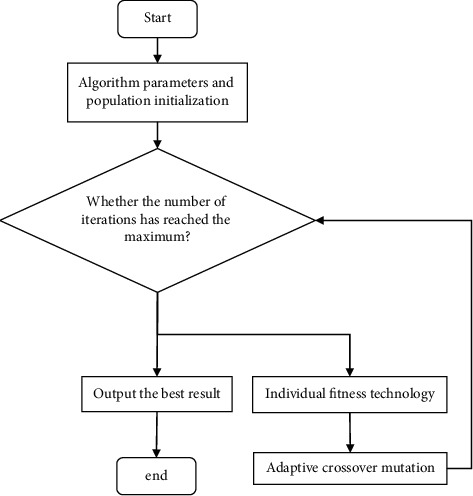
IGA flowchart.

The flowchart of the modified IGA is shown in Figure 1.

## 4. Results

**Figure 2:**
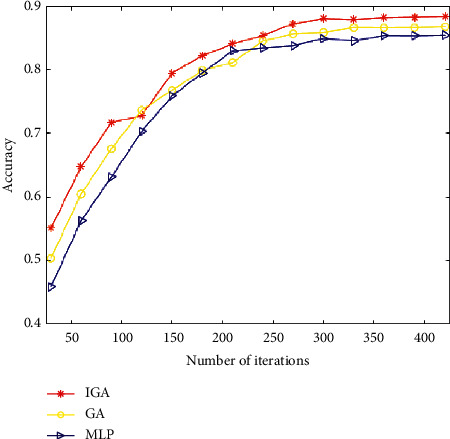
Accuracy as a function of the number of iterations.

We compare and contrast the IGA algorithm, the GA method, and the Multilayer Perceptron algorithm to determine their similarities and differences (MLP).  Figure 2 depicts the variation in the accuracy of the three algorithms as the number of iterations increases. This enables us to conclude that the red line depicts the progression of the IGA algorithm's accuracy as a function of the number of iterations, and that once the number of iterations reaches approximately 300, the accuracy tends to stabilize. It is evident that the GA algorithm achieves a higher level of final accuracy than either of the other two algorithms. The yellow line depicts the change in accuracy of the GA algorithm with the number of iterations, which tends to stabilize when the number of iterations reaches approximately 350. The changing trend of the IGA method and the GA algorithm are virtually identical. However, the GA algorithm's final accuracy is significantly lower than that of the IGA algorithm. The blue line indicates the general progression of the MLP algorithm. From the beginning of the iteration to the very end, we can observe that the algorithm's accuracy steadily improves until it reaches a stable state. In contrast to the other two algorithms, this one yields the least precise results.

**Figure 3:**
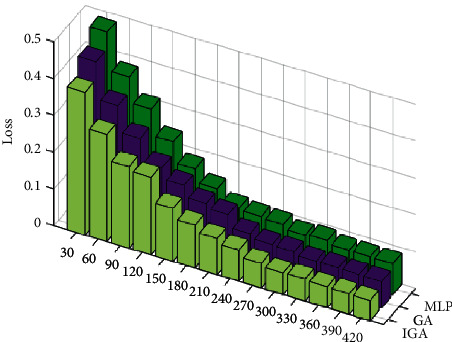
Loss as a function of the number of iterations.

Figure 3 depicts the progression of the three distinct loss values as a function of the number of rounds. It is evident that increasing the number of iterations in any of the three approaches reduces the total loss. The IGA algorithm experiences the smallest initial loss, followed by the GA algorithm, and the MLP algorithm experiences the greatest initial loss. The algorithm MLP causes the largest loss. As the number of rounds increases, the amount of data lost by each of the three algorithms gradually decreases until it eventually stabilizes. The final loss indicates that the IGA algorithm results in the smallest loss, the GA algorithm results in the second-smallest loss, and the MLP algorithm results in the greatest loss.

Additionally, we perform hypothesis testing and *t*-tests on the data of both methods for both metrics and obtain *p*-values less than 0.05, indicating that the method presented in this paper is statistically significantly superior to the traditional method.

**Figure 4:**
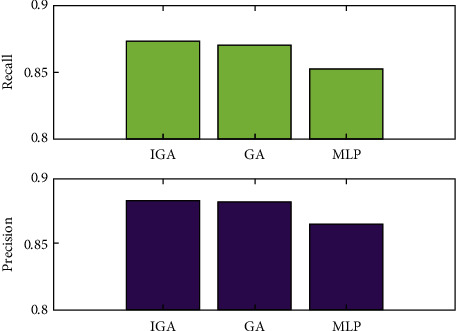
Recall and precision of the three algorithms.

In Figure 4, the recall and precision of the three methods are compared. The recall rate of the IGA algorithm is superior to that of the GA algorithm and the MLP algorithm, as depicted in Figure 4(a). On the other hand, the recall rates of the IGA method and the GA algorithm are not significantly different. Using the chart in Figure 4(b), the accuracy of the IGA algorithm, the GA algorithm, and the MLP algorithm are compared. The IGA algorithm is the most accurate, followed by the GA method, and the MLP algorithm is the least accurate.

**Figure 5:**
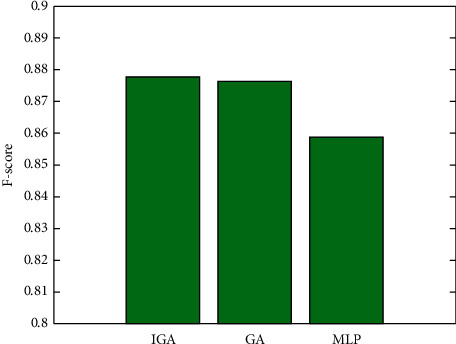
*F*-score of the algorithms.

Figure 5 depicts a comparison chart of the *F* values of the three algorithms. This chart demonstrates that the IGA method has the highest *F* value of the three algorithms, surpassing both the GA and MLP algorithms. The comparison results indicate that the IGA method is superior to the other two algorithms.

## 5. Conclusion

As a result of the advancement of society as a whole, a variety of industries are flourishing, and tourism is making tremendous progress. Compared to the past, a number of regions have made significant strides in the development of tourism, the scale of the industry has continued to expand, the structure of the industrial system has also been steadily improving, and the general population's standard of living has increased dramatically. Due to the increasing number of individuals who choose to pursue some form of spiritual fulfillment, travel has become an essential option for most people's pastimes and relaxation activities. As science and technology continue to advance, the use of big data analysis, algorithms, and other forms of artificial intelligence to calculate and plan a personalized travel route strategy that is suitable for the people will become more convenient for modern tourism. This will reduce the amount of time and money that individuals spend traveling. The progression of events cannot be prevented. In this paper, based on an analysis of the existing problems in tourist route planning, we propose an IGA-based algorithm for optimal round-trip route planning in tourism. This algorithm takes the preferences of individual tourists into account and provides the optimal route options. The shortest path to the next scenic location and iteratively calculating the motivation output value for all interest-characteristic scenic locations based on multiple path interval iteration indicators. Finally, it identifies the optimal travel route that satisfies the psychological needs of tourists in order to maximize motivation and benefit satisfaction. The results of the experiments indicate that the algorithm is consistent with the tourism reality and possesses significant feasibility and practical relevance for intelligent tourism route planning. Both its ability to conduct a local search and the quality of the solutions it generates are improved by the algorithm.

The GA itself is fraught with complications and restrictions. As the problem's complexity increases, the acquired solution cannot be verified, and encoding and decoding operations are frequently problematic. It is highly reliant on the initial population's individual quality. Due to the probability of crossover and the probability of mutation, the algorithm is unable to control its ability to find the optimal solution. In order to maximize the benefits it provides, it must be developed for a variety of real-world application scenarios. This study demonstrates that the application of an improved evolutionary algorithm to the optimization of the ideal tourist route has a high application value, merits further development and promotion, and is applied to the problem of locating a solution for a complex route. The algorithm presented in this work contains additional flaws. This work, for instance, utilizes optimization for a single purpose. In reality, a tourist may have multiple tourism needs, all of which must be maximized simultaneously for the best experience possible. The next line of inquiry will concentrate on locating an efficient solution to the problem of multiobjective optimization in the context of tourism route advice.

## Data Availability

The data used to support the findings of this study are available from the corresponding author upon request.

## References

[1] Ma M., Cai W. (2019). Do commercial building sector-derived carbon emissions decouple from the economic growth in Tertiary Industry? A case study of four municipalities in China[J]. *Science of the Total Environment*.

[2] Zong J., Ding Z. Feedback-based coarse time-granularity POI recommendation and itinerary planning.

[3] Hsu F. C., Chen P. (2000). Interactive genetic algorithms for a travel itinerary planning problem[J]. *TSP*.

[4] Singhal A., Jindal B. K. GoTrip: an automatic ontological travel itinerary planner using SPARQL inferencing.

[5] Mehmood G., Zahid Khan M., Fayaz M., Faisal M., Ur Rahman H., Gwak J. (2022). An energy-efficient mobile agent-based data aggregation scheme for wireless body area networks. *Computers, Materials & Continua*.

[6] Dunstall S., Horn M. E. T., Kilby P. (2003). An automated itinerary planning system for holiday travel[J]. *Information Technology & Tourism*.

[7] Jünger M., Reinelt G., Rinaldi G. (1995). The traveling salesman problem[J]. *Handbooks in Operations Research and Management Science*.

[8] Hoffman K. L., Padberg M., Rinaldi G. (2013). Traveling salesman problem[J]. *Encyclopedia of operations research and management science*.

[9] Flood M. M. (1956). The traveling-salesman problem. *Operations Research*.

[10] Mirjalili S. (2019). Genetic algorithm[M]. *Evolutionary Algorithms and Neural Networks*.

[11] Whitley D. (1994). A genetic algorithm tutorial[J]. *Statistics and Computing*.

[12] Kumar M., Husain D., Upreti N. (2010). Genetic algorithm: review and application[J]. https://papers.ssrn.com/sol3/papers.cfm?abstract_id=3529843.

[13] Mathew T. V. (2012). Genetic algorithm[J].

[14] Sakoe H., Chiba S. (1978). Dynamic programming algorithm optimization for spoken word recognition. *IEEE Transactions on Acoustics, Speech, & Signal Processing*.

[15] Van Laarhoven P. J. M., Aarts E. H. L. (1987). Simulated annealing[M]. *Simulated Annealing: Theory and Applications*.

[16] Dorigo M., Birattari M., Stutzle T. (2006). Ant colony optimization. *IEEE Computational Intelligence Magazine*.

[17] Logesh R., Subramaniyaswamy V. (2019). Exploring hybrid recommender systems for personalized travel applications[M]. *Cognitive Informatics and Soft Computing*.

[18] Renjith S., Sreekumar A., Jathavedan M. (2020). An extensive study on the evolution of context-aware personalized travel recommender systems. *Information Processing & Management*.

[19] Nitu P., Coelho J., Madiraju P. (2021). Improvising personalized travel recommendation system with recency effects. *Big Data Mining and Analytics*.

[20] Coelho J., Nitu P., Madiraju P. A personalized travel recommendation system using social media analysis[C].

[21] Majid A., Chen L., Chen G., Mirza H. T., Hussain I., Woodward J (2013). A context-aware personalized travel recommendation system based on geotagged social media data mining. *International Journal of Geographical Information Science*.

[22] Dao T. H., Jeong S. R., Ahn H. (2012). A novel recommendation model of location-based advertising: context-Aware Collaborative Filtering using GA approach. *Expert Systems with Applications*.

[23] Zhou D., Wang B., Rahimi S. M. A study of recommending locations on location-based social network by collaborative filtering[C].

[24] Lee C. K. M., Yeung C. L., Xiong Z. R., Chung S (2016). A mathematical model for municipal solid waste management – a case study in Hong Kong. *Waste Management*.

[25] Mladenović N., Hansen P. (1997). Variable neighborhood search. *Computers & Operations Research*.

[26] Hansen P., Mladenović N. (2001). Variable neighborhood search: principles and applications. *European Journal of Operational Research*.

[27] Pettie S., Ramachandran V. (2002). An optimal minimum spanning tree algorithm. *Journal of the ACM*.

